# Recent Patents of Interest to Dentists

**Published:** 1906-08

**Authors:** 


### RECENT PATENTS OF INTEREST TO DENTISTS.

824087, Tooth brush, Solon E. Babcock, Plateau City, Colo.
824465, Metallic support for artificial teeth, David N. Booth, New York.
824096, Dental articulator, Walter W. Crate, Camden, N. J.
824111, Making teeth, Harry A. Gollobin, Newark, N. J.
824218, Making dental devices, Stanley Towle, Fall River, Mass.
825268, Antiseptic and perfumed block for dental stoppings and other similar purposes, Lucien Eilertsen, Paris, France.

825271 ,Right-angle bur-mandrel, George N. Guthrie, Jr., Cookeville, Tenn.

825578, Tooth-shade guide, Arthur W. Browne, Prince Bay, New York.

825356, Artificial tooth for crowns and bridges, Wm. B. Short, New York.

825891, Dental engine, Arthur W. Browne, Prince Bay, N. Y.

825896, Dental measuring tool, Francis X. Dusseau, and B. F. Kirk, Detroit, Mich.

8825901, Vulcanizer, Edmund D. Gilbert, Philadelphia, Pa.

825940, Crown-tooth attachment, Henry H. Schuhman, Hartford, Wis.

Copies of the above patents may be obtained for ten cents each, by addressing John A. Saul Solicitor of Patents, Fendall Building, Washington, D. C.

